# Self-regulation of emotional responses to Zika: Spiral of fear

**DOI:** 10.1371/journal.pone.0199828

**Published:** 2018-07-10

**Authors:** James Price Dillard, Chun Yang, Ruobing Li

**Affiliations:** 1 Department of Communication Arts & Sciences, Pennsylvania State University, University Park, Pennsylvania, United States of America; 2 Manship School of Mass Communication, Louisiana State University, Baton Rouge, Louisiana, United States of America; Universitat Wien, AUSTRIA

## Abstract

Fear of infectious disease can create a variety of problems not the least of which is fear itself. An important question is how individuals attempt to manage their fear. The appearance of Zika in the U.S. presented an opportunity to examine this issue in a consequential natural context. Beginning nine days after the W.H.O. declared Zika a world health crisis, two-waves of survey data were collected from women ages 18–35 who were living in the Southern U.S. (*N* = 561). Most respondents (71%) used one or more emotion regulation strategies and a plurality (41%) utilized multiple strategies. Fear of Zika showed no demonstrable effect on avoidance, reappraisal, or contesting and none of these three strategies were effective at down-regulating fear. Fear and suppression, however, showed a self-reinforcing cycle in which fear increased use of suppression and suppression increased intensity of the fear response. Although the observed associations were small, even modest effects can be consequential when cumulated over time or across large numbers of individuals.

## Introduction

Fear of infectious disease is responsible for a plethora of problems including acceleration of the spread of disease [1], delays in care-seeking [2], disruption of health-care delivery systems [3], and economic downturns [4]. A point less widely appreciated, but no less true, is that fear creates harm at the individual level as well. In addition to simply being unpleasant, it may interfere with the ability to perform one’s job or to successfully enact social relationships, such as that of parent or spouse [5]. Fear has also been associated with diminished cardiovascular health [6], decreased immune functioning [7] and degraded psychological well-being [8].

Given its importance to public health at both the social and individual levels, it is surprising that so little is known about how individuals attempt to self-regulate fear. The appearance of Zika in the U.S. presented an opportunity to examine this issue in a consequential natural context. The research focused on three interlocking questions: (a) To what extent do individuals utilize emotion regulation strategies in response to a health crisis? (b) Which strategies are instigated by fear? and (c) Are those strategies effective at down-regulating fear? Before examining these issues, it is important to understand the context created by the World Health Organization’s (WHO) declaration of Zika as an international health emergency.

### Context

**Fig 1:**
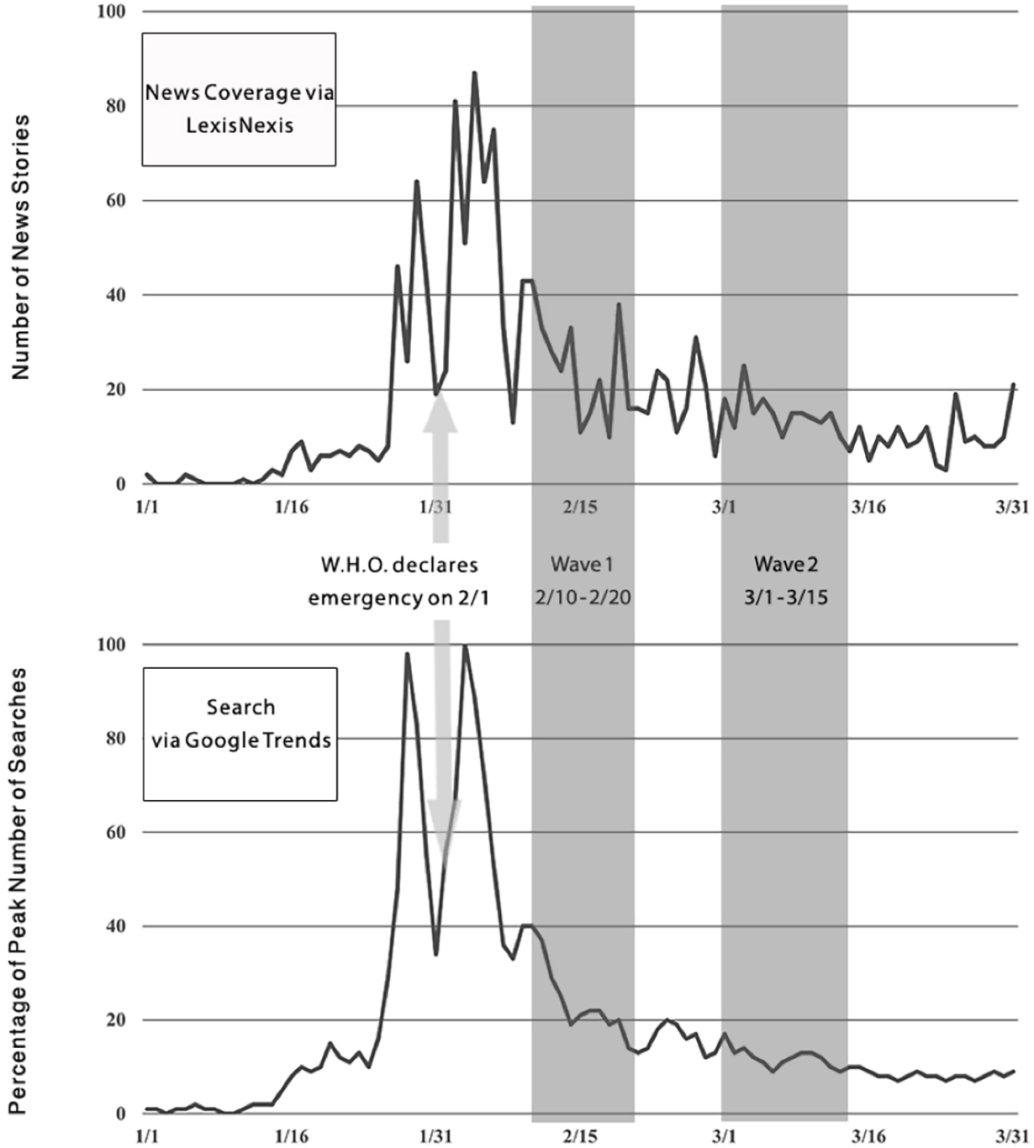
Zika-related media coverage and information search in early 2016. Google Trends is a public web facility that shows how frequently a search-term is entered relative to the total search-volume for that term in specifiable regions of the world. Fig 1 shows the results for the “Zika” in the U.S. during the period January 1 to March 31. Similar to the newspaper data, the Internet search results show spikes of activity just before and after the WHO declaration of crisis. As with the news coverage data, search behavior shows a general downward trend over the time period of interest, but one that is marked by considerable fluctuation around the overall direction. Together the two data displays make the case that the media environment during early 2016 was both dynamic and highly variable. This was the context in which our study of Zika-related fear took place.

A great deal of health-related information reaches the public via the mass media, Internet search, or both. To gain insight into the media environment in early 2016, we conducted a Lexis/Nexis search for the word “Zika” in U. S. newspapers for the period January 1 to March 31. As can be seen in Fig 1, news coverage was intermittent at the beginning of the year, but it increased almost 90-fold by mid-February. In terms of content, there was speculation regarding Zika-induced brain damage in infants as early as December, 2015. The same stories often reported the possibility of Guillain-Barré syndrome in adults [9]. By early January, health experts were expressing suspicion that the virus could be transmitted by sex as well as mosquitos. As with other health crises (e.g., Ebola), news coverage often expressed alarm. In one article, the interviewee, Dr. Schaffner, described Zika as “enormously anxiety-provoking” [10]. On February 1st, the WHO declared Zika an international health emergency [11].

### The nature of emotion

Emotions are functionally coherent patterns of activation across multiple biopsychosocial systems. Much current theory holds that emotions are the product of evolutionary design [12]. Their purpose is to, first, identify particular configurations of person-environment relationships, then to enable behaviors suitable to managing those relationships. Fear may be considered to be a state that intended to produce an effective behavioral response to a perceived threat. The threat may be perceived directly such as when a driver sees an oncoming automobile drift across the yellow line and into her lane. However, judgments of threat may also arise indirectly from warnings issued by (usually) members of the same species. A passenger might alert a driver of about a dangerous curve that lies ahead. Media or government might warn the public about downed power lines across a section of highway.

Preparation for behavior includes changes in the cognitive, physiological, expressive, motivational, and subjective systems. The cognitive system manifests heightened vigilance, especially toward the perceived threat, and biases in reasoning such as a tendency to overestimate the likelihood of negative outcomes [13]. In the physiological domain, fear is associated with heightened startle reflex, greater heart rate reactivity, and increased sweating [14]. Facial display of fear is characterized by eyebrows that are raised and pulled together, raised upper eyelids, tensed lower eyelids, and lips slightly stretched horizontally back to the ears [15]. Motivationally, fear is associated with behavioral inhibition [16], presumably for the purpose of distancing the organism from the threat. Finally, fear is experienced as a negatively valenced state indexed by words such as *afraid* and *scared*. The subjective state is not merely an ephemera. Rather, it represents a summary signal of changes across multiple systems [17]. That overall pattern of changes is represented in consciousness as the experience of fear.

The general function of emotion is to prioritize a problem-specific set of actions over other ongoing behaviors. This is useful to the extent that the threat is urgent and solvable, but, when the threat is open-ended and there is no effective remedy, constant prioritization is dysfunctional. Thus, individuals may seek to down-regulate fear for instrumental reasons [5]. Of course, fear is also subjectively aversive. Individuals may attempt to lessen their fear because the experience of it is disagreeable [5].

### Emotion regulation

Emotional regulation is the effort to modulate emotional experience in terms of the valence, intensity, or duration of the affect. Generally, individuals strive to manage their emotions in a pro-hedonic manner, that is, by minimizing negative affects and enhancing or maintaining positive affects. There are, however, exceptions in which individuals seek to decrease positive affect (e.g., suppressing laughter at a funeral) or increase negative affect (e.g., increasing anger prior to a confrontation). Because we cannot readily imagine any motivation for counter-hedonic regulation of Zika-related fear, the assumption that individuals would attempt to down-regulate their fear guided our thinking. Prior research suggested several strategies that might be utilized in an effort to achieve that goal [18,19]:

#### Avoidance

Following initial contact with a frightening topic, individuals may decide to proactively evade individuals or situations that might re-expose them to the issue [20, 21]. Psychologists such as Gross [18] refers to this as *situation selection*, whereas media researchers, such as Knobloch-Westerwick & Meng [22] label it *selective exposure*.

#### Reappraisal

This family of cognitive change strategies has several members including hopelessness (“I might as well just give up”), fatalism (“There is nothing that I can do”) and risk normalization (“Everything has some risk associated with it”). [23] Common themes among the various forms of reappraisal are passivity, acceptance, and distancing one’s self from the emotional issue.

#### Contesting

This involves actively arguing against claims of a hazard [24]. Like reappraisal, contesting can take several forms including denial of the severity of the threat, rejection of susceptibility to the threat, or derogation of the message source or the issue [21].

#### Suppression

We use this term to mean the conscious exertion of mental effort to subdue unwanted thoughts or feelings [25, 26]. It occurs when people try to tamp down, put away, or compartmentalize dispreferred mental activities.

It is surprising that relatively little attention has been given to basic descriptive questions about emotional regulation strategies such as the frequency with which these strategies are used [27, 28]. This is especially true with regard to how individuals respond to threatening events such as health crises. To move toward filling this gap in knowledge, we asked what proportion of individuals were engaged in emotional regulation:

RQ1: How widely used are each of the emotion regulation strategies following declaration of an international health crisis?

### The effect of fear on regulation strategies

**Fig 2:**
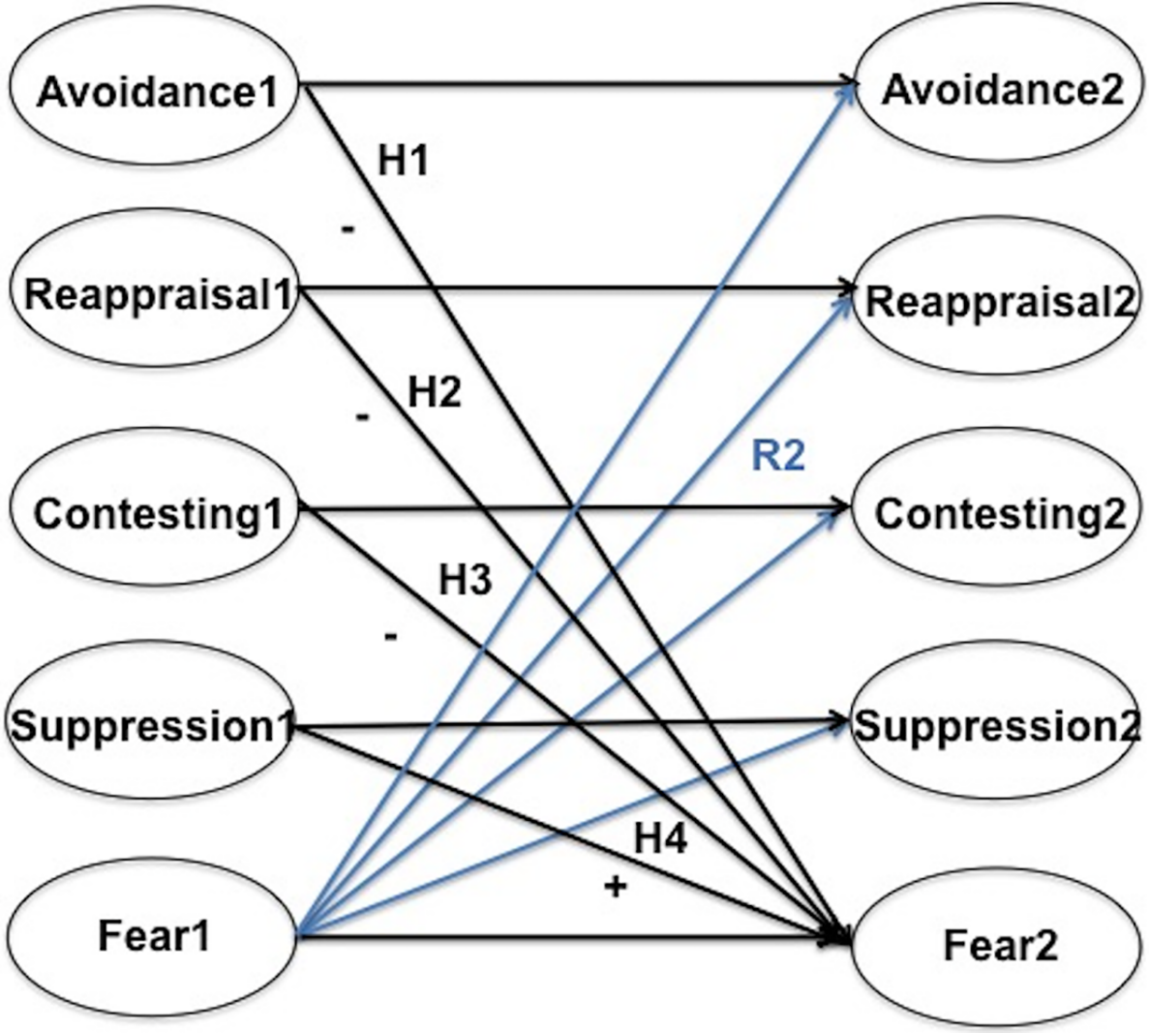
The initial model. Not shown are the correlations among the exogenous variables and the correlated item residuals across waves.

Emotion is the causal antecedent of regulation: Without the occurrence of emotion there would be nothing to regulate. In experimental studies of emotional regulation participants are often trained in a particular strategy, then encouraged to use it when exposed to the stimulus [19]. This may be contrasted with real-world circumstances in which individuals are left to their own devices where they may choose one strategy or several. Accordingly, we posed another basic, descriptive question (represented graphically in Fig 2).

RQ2: To what extent does fear of Zika cause the use of avoidance, reappraisal, contesting, or suppression, alone or in combination?

### The effect of regulation strategies on fear

It has been argued that avoidance is likely to be among the most effective means of emotional regulation because stopping something once it is underway is generally more difficult than preventing it from occurring in the first place [29]. By this logic, we anticipated that:

H1: Avoidance is negatively associated with fear of Zika.

Webb et al.’s [19] meta-analysis reported medium-sized, positive effects for reappraisal on self-report and behavioral indices of emotion (i.e., effective down-regulation). Presumably, the distancing brought about by reappraisal reduces involvement with the stimulus, which is a necessary condition for emotional arousal [30]. Accordingly, we expected that:

H2: Reappraisal is negatively associated with fear of Zika.

With regard to infectious disease, a correlational study of emotional response to Ebola found that one form of contesting–derogation of message sources–was an effective means of down-regulating fear [31]. Because this result was compatible with proposals to understand contesting as a form of defense [32], we hypothesized that:

H3: Contesting is negatively associated with fear of Zika.

Some experimental investigations indicate that suppression is a counterproductive strategy in that it leads to heightened negative emotional experience [33, 34]. The meta-analytic data are less consistent: Webb et al. [19] found no reliable effect of suppression on self-reported emotions, but they did observe a small negative relationship with physiological indices. Ironic process theory suggests that the failure of suppression is all but inevitable because the “processes that undermine the intentional control of mental states are inherent in the very exercise of such control” (p.34) [35]. A body of work supports that contention in various contexts [35] including cross-sectional evidence that suppression is positively associated with fear of Ebola [31]. Thus, we expected that:

H4: Suppression is positively associated with fear of Zika.

### Personal relevance of the threat of Zika

Theories of emotion assert that affect follows from a configuration of appraisals focused on the relationship between the individual and the environment. One such appraisal is change, a condition that is met whenever individuals become aware of a new disease. Yet, even though a population may be uniformly aware of a change, Zika does not present an equal risk to all of its members. Women who are currently pregnant, or planning to become pregnant in the near future, bear a disproportionate risk due to the possibility of microcephaly in the infant. Assuming that they are aware of this danger, it is possible that fear and emotion regulation are more tightly intertwined than for those who are not pregnant and do not plan to become pregnant. Accordingly, we asked:

RQ3: Are associations between fear and emotion regulation stronger among women for whom Zika is high (vs. low) in personal relevance?

## Materials and methods

### Procedures and participants

Via the Qualtrics national online survey panel (http://www.qualtrics.com), we initially recruited 1002 women between the ages of 18 and 35 who resided in states that border Mexico (Arizona, New Mexico, and Texas) or have a Caribbean shoreline (Texas, Louisiana, Mississippi, Alabama, and Florida). California was not included because such a large portion of the state is outside of the range of the mosquitoes thought to carry the disease. Because the panel is opt-in, it is not possible to calculate a response rate (given that the denominator for the ratio is unknown). The investigation was approved by the Institutional Review Board at The Pennsylvania State University. The first page of the survey described the study and consent was indicated by forward movement through the survey.

Our first wave of data collection began February 10th, nine days after WHO declared Zika an international health crisis, and lasted until February 20th. The second wave took place from March 1st-15th (Fig 1). The average time between survey responses was 10 days with a range of 8 to 32 days. Due to a recording error during data collection, 90 members of the wave 1 sample were not re-contactable. Hence, the wave 2 *N* of 561 represents a (561/912 =) 62% retention rate.

### Statistical power

Assuming N = 561 and a two-tailed test, power to detect a bivariate effect equivalent to *r =* .10 was .77. For effect sizes of .20 and above, power was greater than .99 [36].

### Missing data

Although participants could decline to answer any question, there were no missing data on any of the variables included in this paper.

### Measures

Items for the measurement of fear and emotional regulation strategies were based on previous research [31]. Fear was assessed with this stem: *For each of the words below*, *please mark the response that best represents how the current news about Zika makes you feel*. Two items (*scared* and *afraid*) were presented and accompanied by a 5-point response scale that ranged from 0 = none of this feeling to 4 = a great deal of this feeling.

Four items were written to assess each of the emotional regulation strategies. Strategies and sample items were: Avoidance (*I actively avoided news about Zika* and *I avoided situations where I would hear about Zika*), reappraisal (*I remembered that life is full of risks*: *You have to accept that fact* and *I reminded myself to accept that which I cannot change*), contesting (*I remembered that most of what we are hearing about Zika is blown out of proportion* and *I reminded myself that people are making too big a deal out of Zika*) and suppression (*I made an effort not to think about Zika* and *I tried to tamp down my feelings about Zika*). The five-point response scale was 1 = strongly disagree, 2 = disagree, 3 = neither agree nor disagree, 4 = agree, and 5 = strongly agree.

In the first wave only, two dichotomous-choice items assessed the personal relevance of Zika (*I expect that I will become pregnant in the next two years* and *I am pregnant now*). Women who responded affirmatively to either or both items were assigned to the high personal relevance group (*N* = 234) and those who responded negatively to both were assigned to the low personal relevance group (*N* = 327). The full survey is given in S1 Survey.

### Plan for analysis

Following item level analyses, items were submitted to exploratory and confirmatory factor analyses. The resulting scales were analyzed via longitudinal structural equation modeling to assess the questions of theoretical interest. The measurement and theoretical models were evaluated in terms of their fit to the data using the following guidelines for preferred values: χ^2^/df < 3 [37], Tucker-Lewis Index (TLI) > .94, standardized root mean residual (SRMR) < .08, root mean squared error of approximation (RMSEA) < .08, and the probability of close fit, PCLOSE > .05 [38,39].

## Results

### Measurement model

To explore the structure of the data, a principal axis factor analysis was run on the full wave 1 data (N = 1002). This produced five factors whose eigenvalues were greater than 1 and whose interpretation matched expectations (i.e., one fear factor and four regulation factors). After eliminating items that showed poor loadings (< .60) on their intended factors or cross-loadings (>.40) on unintended factors, 14 items remained (three for avoidance, two for reappraisal, four for contesting, three for suppression, and two for fear). A second principal axis analysis on these items produced a clean five-factor solution.

Confirmatory procedures were then used to test the five-factor model for measurement invariance in the two-wave data set. The ten latent variables were allowed to correlate with one another within and between waves. To model item-specific variance, the error terms for items in the first wave were permitted to correlate with their partners in the second wave. Following the procedures outlined in Little [40], we estimated configural invariance, loading invariance, and intercept invariance in that order. The final and most restrictive model showed good fit to the data: χ^2^ (319) = 585.96, *p* < .0001, χ^2^/df = 1.83, TLI = .972, SRMR = .033, RMSEA = .039 (90% CI. = .034/.044), and PCLOSE = 1.00. No pairwise comparison of the CFIs was greater than .01, which is the criterion suggested by Cheung and Rensvold [41]. Thus, the measurement model met the criteria for strong invariance.

### Preliminary analyses and RQ1

Tables 1 and 2 present the correlations among the variables and their corresponding descriptive statistics in waves 1 and 2, respectively. Of special note, associations among the regulation strategies are substantial and positive in both waves, a pattern that indicates that individuals who use any one strategy are prone to employ others as well.

**Table 1:** Bivariate correlations and descriptive statistics for manifest variables in (mostly) wave 1.

	1	2	3	4	5	6
1. Avoidance@w1	.90					
2. Reappraisal@w1	.22*	.70				
3. Contesting@w1	.54*	.36*	.91			
4. Suppression@w1	.66*	.41*	.54*	.84		
5. Fear@w1	.008	.05	-.13*	.11*	.89	
6. Fear@w2	.10*	.03	-.002	.17*	.53*	.90
Mean	2.40	3.43	2.96	2.81	2.11	1.87
SD	1.02	.88	.97	.94	1.12	1.21

*N* = 561. Diagonal entries are alpha coefficients. Waves 1 and 2 indicated by w1 and w2, respectively.

*p < .05.

**Table 2:** Bivariate correlations and descriptive statistics for manifest variables in (mostly) wave 2.

	1		2	3	4	5	6
1. Avoidance@w2	.94						
2. Reappraisal@w2	.21*		.76				
3. Contesting@w2	.49*		.41*	.92			
4. Suppression@w2	.60*		.44*	.45*	.85		
5. Fear@w1	.003		.07	-.05	.18*	.89	
6. Fear@w2	.08*		.04	-.06	.26*	.53*	.90
Mean	2.38		3.44	2.99	2.80	2.11	1.87
SD	1.05		.91	.97	.94	1.12	1.21

*N* = 561. Diagonal entries are alpha coefficients. Waves 1 and 2 indicated by w1 and w2, respectively.

*p < .05.

RQ1 asked about the extent to which respondents used each of the regulation strategies. To address that question we created a binary variable for each strategy that broke its data at the scale midpoint. Given the 1-to-5 response scale, values greater than 3 indicated that respondents used the strategy, whereas values of 3 or less indicated uncertainty or non-use. For avoidance, 19% of the sample fell above the midpoint at time 1 and 19% at time 2. The corresponding values for reappraisal were 57% and 58%, for contesting 40% and 40%, and for suppression 31% and 31%.

To assess RQ2, we conducted a count of the number of regulation strategies used by any given individual. For time 1, 29% of the sample used no regulation strategies, 30% used one strategy, 18% used two strategies, 12% used three strategies, and 11% used all four strategies. The corresponding data for time 2 were: none (29%), one (28%), two (18%), three (13%), and four (11%). Overall, a majority of our sample (71%) used one or more emotion regulation strategies and a plurality (41%) utilized multiple strategies. In addition to their substantive importance, these results indicated that there was sufficient variation in the strategy use to carry out the next set of analyses.

### Theoretical model and RQ2, H1-H4

The initial analysis tested the causal model shown in Fig 2 with two additions: The exogenous variables were allowed to correlate and the error terms for individual items were allowed to correlate with themselves across waves. Although some of the fit statistics were acceptable, the overall pattern was not optimal (see Table 3). Inspection of the modification indices revealed that fit could be improved by allowing the errors of prediction to correlate. Substantively, this means that the model is incomplete: One or more variables that affect the system of equations is missing. Given the rapidly changing communication environment during the survey period (Fig 1), this seemed a plausible assumption. On the premise that the rationale for allowing any pair of errors to correlate should be implemented in all corresponding circumstances, we permitted correlations among all of the errors of prediction. The results are given in Table 3 in the column labeled Model 2. The next column, Model 3, shows the results after removal of nonsignificant paths from Model 2. With the exception of the highly sensitive χ^2^ test, all of the coefficients indicated good fit. Thus, we turned to interpretation of the parameter estimates.

**Table 3:** Theoretical models of fear and emotional regulation.

	Model 1	Model 2	Model 3
Fit Indices	correlated exogenous variables, correlated item residuals across waves, autoregressive paths, hypothesized paths	Model 1+correlated disturbance terms	Model 2 trimmed of nonsignificant paths
χ^2^ (df)	960.81 (313)***	545.64 (303)***	549.08 (309)***
χ^2^/df	3.07	1.80	1.77
TLI	.93	.97	.97
SRMR	.087	.037	.038
RMSEA (90% ci)	.061 (.056/.065)	.038 (.033/.043)	.037 (.032/.042)
PCLOSE	.000	1.00	1.00

χ2 = chi-squared statistic. df = degrees of freedom. TLI = Tucker-Lewis Index. SRMR = Standardized root mean residual. RMSEA = root mean squared error of approximation. PCLOSE = probability of close fit.

*p < .05

**p < .01

***p < .001.

RQ2 was concerned with the influence of fear on emotional regulation strategies. The standardized coefficients were .00, *p* = .99 for avoidance; .05, *p* = .23 for reappraisal; .03, *p* = .37 for contesting; and .13, *p* = .002 for suppression. Hence, the evidence indicated that fear encouraged the use of suppression, but showed no demonstrable influence on the other three regulation strategies.

The hypotheses focused on the effects of regulation strategies on emotional intensity. H1 predicted a negative association between avoidance and fear. Because the standardized path coefficient was -.03, *p* = .70, H1 was not supported. H2 predicted a negative association between reappraisal and fear, but the coefficient of -.07, *p* = .22 gave no support to H2. H3 anticipated that contesting would exhibit a negative influence on fear. Given a path coefficient of .01, *p* = .81, H3 was not supported. The fourth hypothesis predicted a positive association between suppression and fear. The coefficient of .18, *p* = .050 was taken as consistent with H4.

**Fig 3:**
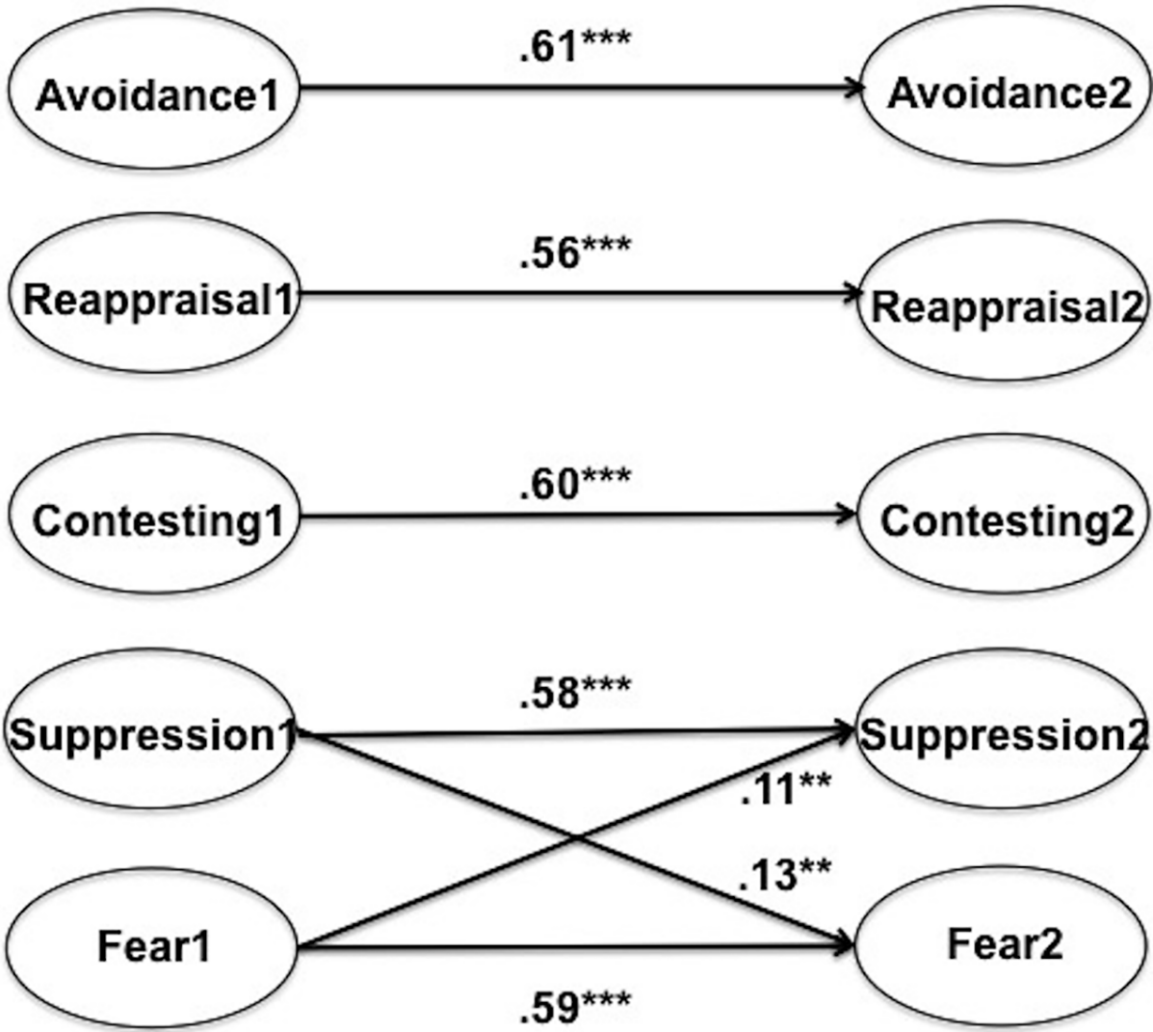
Final model with standardized regression weights. Not shown are correlations among the exogenous variables, correlations among the disturbance terms, or correlated item residuals across waves. *p < .05. ** *p* < .01. *** *p* < .001.

To complete the analyses, we trimmed the non-significant paths, then re-estimated the parameters in Model 3. In terms of fit statistics, the difference between Models 2 and 3 was miniscule. Further, all of the retained parameters were significant at *p* = .002 or better (including the suppression@t1-fear@t2 association). Fig 3 displays the final obtained model and the standardized parameter estimates. There are two notable features of the results. First, there was a self-reinforcing relationship between suppression and fear: Fear increased the use of suppression and suppression increased the experience of fear. Second, there was no indication that any of the emotional regulation strategies were either caused by fear nor that they were effective at down-regulating fear.

### Theoretical model and RQ3

Multi-group structural equation analyses were conducted to assess the operation of the obtained theoretical model in the high and low relevance groups. These groups were, respectively, participants who were currently pregnant *or* planning to become pregnant (high relevance) versus those not currently pregnant *and* not planning on becoming pregnant (low relevance). Assuming the unconstrained model to be correct, requiring equal measurement weights produced the following: χ^2^ (18) = 27.51, *p* = .070, values that are close to, but do not exceed the conventional *p* < .05 probability level. Next, assuming the measurement weights to be correct, we tested for invariance of measurement intercepts. Not surprisingly, the results indicated group differences: χ^2^ (28) = 64.98, *p* = .0001. Intercept values for fear and the emotional regulation strategies were lower in the low relevance group than in the high relevance group. The third and final comparison addressed RQ3. Assuming the measurement intercepts to be correct, it tested whether the structural weights differed across groups. The results indicated that they did not: χ^2^ (7) = 7.80, *p* = .350.

## Discussion

### Use of emotional regulation strategies

Studies of emotional regulation in natural settings are relatively rare and, to date, have assessed events that occur in day-to-day life [28, 42]. Our focus, in contrast, was on a single, potentially consequential topic. Despite this notable difference, our results mirror some of the earlier findings. For example, all four strategies under study were used by some portion of our sample (19% to 58%). Further, where Heiy and Cheavens reported that individuals in their study used an average of seven strategies to manage fear, our data showed averages of 1.5 and 1.9 in waves 1 and 2 respectively (Values reported here are slightly higher than those in our results section because they exclude individuals who did not report experiencing fear, as was done in the Heiy & Cheavan’s study.) The studies concur that individuals tend to use multiple emotion regulation strategies.

It might also be thought that individuals find a strategy that they like, then stick with it. Indeed, our data also showed that the latent stability coefficients (i.e., the auto-regressive paths) ranged from a high of .64 for avoidance to a low of .56 for suppression. These values are substantial enough to imply considerable stability, but not so high as to indicate that strategy use at one time guarantees use of that strategy at another time. We come to the same conclusion that Brans et al. drew from their intra-class correlations: Strategy use in any given instance is a function of situational *and* personal factors. A comprehensive list of those factors is surely something toward which research should aspire.

### Suppression and the spiral of fear

As Fig 3 shows, suppression and fear were mutually reinforcing over the period of our survey. Fear at time 1 caused suppression at time 2, and suppression at time 1 caused fear at time 2. This pattern of data can be interpreted as evidence of an ironic process [35]. The theory holds that mental control is the product of two interdependent activities. The *operating process* attempts to load consciousness with cognitions and feelings that are relevant to the desired state or, in this case, to eliminate the unwanted experience of fear. The *monitoring process*, which runs below conscious awareness, evaluates whether the operating process is successful and, if not, re-initiates the operating process. Re-initiation has the effect of sensitizing the mind to content that is indicative of system failure. The current application is complicated somewhat by the fact that the desired state is a negation (i.e., not fear). That said, the experience of fear is clear evidence that suppression is failing to eliminate fear. This should be sufficient to restart the operating system, which will instigate further attention to the undesired feeling, thereby resulting in a spiral of fear.

We need not conclude, however, that this spiral is completely self-contained. In fact, it seems likely that factors external to ironic process theory make some contribution to the maintenance of fear. As Fig 1 makes plain, Zika was the topic of a great deal of media coverage and search behavior. And our previous research shows that media coverage was complemented by frequent interpersonal discussions of the disease [43]. Hence, one factor that could recreate fear as the contents of mind is repeated exposure to the threatening concept. Concept exposure can occur for active reasons, as when individuals planfully seek out relevant information, or passive reasons, as when they are inadvertently exposed to news or talk of the disease, perhaps even when they are working to avoid contact. A better understanding of the causes of the fear spiral might yield insights into how that vicious cycle could be disrupted.

### Avoidance, reappraisal, and contesting

The results indicate that we must consider two distinct knowledge claims with respect to avoidance, reappraisal, and contesting. There was no indication that (a) fear prompted the use of any of the three emotion regulation strategies nor that (b) any of these three strategies had an observable effect on fear intensity. There are several methodological issues that are relevant to both knowledge claims. For instance, because the measures showed substantial variance in both waves (Tables 1 and 2), the lack of covariation cannot be attributed to restriction in range. And given our sample size, it is unlikely that the lack of correlation–in either causal direction–is the result of insufficient statistical power, unless the true effects are quite a bit smaller than *r* = .10.

One potential explanation for the null findings resides in the lag time between waves 1 and 2, which was an average of 10 days. Identification of the proper lag is a classic problem in longitudinal research that can be solved by empirical studies devoted to that problem, preferably in conjunction with the development of theory that explicitly articulates the temporal dynamics of emotional regulation [40]. Having no such theory in hand, our procedures were guided primarily by pragmatic concerns, the most central of which was the speed at which respondents accrued. However, it is important to note that to the extent that the process of emotion regulation is non-recurrent and faster than our lag, the observed effect will be downwardly biased [44]. If that is true in our data, it would explain the apparent absence of effects for avoidance, reappraisal, and contesting. It would also suggest that even the coefficients that were significant are smaller than they would be in a study with a shorter lags. Perhaps an alternative method such as experience sampling could be used in future work to capture and track emotional response and regulation at a faster rate.

An alternative explanation for the null findings resides in our approach to analysis. Much research on emotion regulation is experimental, a method that enables strong causal inference, but also demands simplicity. Participants are typically assigned to use a single emotion regulation strategy, whose effectiveness is then assessed against other conditions/strategies. Our analytic approach differed from this in that it controlled for autoregressive influences (i.e., effects of variables on themselves) as well as the simultaneous effects of other regulation strategies [42]. There is no doubt that explicitly modeling these influences had an effect on our conclusions. In preliminary regression analyses that ignored autoregressive effects, contesting (t1) showed the anticipated negative effect on fear (t2). And as can be seen in the correlations in Tables 1 and 2, avoidance (t1) has a bivariate effect on fear (t2), which vanished when accounting for suppression (Fig 2). By failing to explicitly model these effects, the experimental literature may be overestimating the degree of association between regulation strategies and emotional response, a point made previously by Brans et al. [27].

### Personal relevance

The threat of Zika was more or less personally relevant to the women in our sample as a function of the pregnancy status and pregnancy intentions. However, there was no indication that this difference influenced the pattern of associations between fear and the emotional regulation strategies. This does not mean than personal relevance is unimportant to understanding emotional arousal and regulation. Indeed, the mean levels of fear were higher in the high relevance group: 2.39 versus 1.90, *t* (559) = 5.32, *p* = .0001 at time 1 and 2.09 versus 1.71, *t* (559) = 3.72, *p* = .0001 at time 2. However, the structural equation analyses highlight a different aspect of the data, that is, the ordering of values between the two groups. These results lead to the conclusion that the ordering, and the resulting associations, are robust across levels of personal relevance. In other words, the processes are the same in both groups, even though the mean levels are not.

### Context specific findings and implications

Although our comments thus far have focused on basic theoretical and methodological issues, it is important not to overlook implications of the findings for applied questions of public health. Several observations are in order. For one, it is clear that the women in our sample were frightened. Ninety percent of the wave 1 sample gave a non-zero answer when asked if they were fearful given the current news about Zika. The corresponding value for wave 2 was 85%. Nine percent and 8% reported the highest possible scale value in waves 1 and 2 respectively.

To the extent that fear is damaging to the individuals who experience it, these values may be worrisome. It is of particular concern that fear in mothers can negatively impact the well-being of infants. To this point, Lederman et al. [45] reported that physical proximity to the World Trade Center (a proxy for fear) was associated with reduced birth weight of infants who were *in utero* at the time of the 9/11 attacks. Comparison of birth weights among Dutch neonates whose mothers were exposed to the attack only via media versus a cohort assessed a year later also showed reduced birth weight [46]. It would be ironic if fears about infant well-being, such as microcephaly, were themselves the cause of diminished infant health.

Interventions designed to reduce fear intensity need to consider two distinct goals. One is to encourage dismissal of counterproductive regulation strategies, that is, suppression. Of course, the design of such an intervention is contingent on first understanding why nearly one-third of our sample elected to employ this strategy, then persisted in its use at time 2 despite having personal evidence of its ineffectiveness.

The second goal of an intervention should be to promote approaches to emotion regulation that are simultaneously effective and realistic. Because none of the strategies assessed in this research provided relief from fear, it may be necessary to search for new techniques or to develop efficacious variations on known strategies. Tests of alternatives will need to be conducted in circumstances similar to our study, where uncertainty about disease outcomes is high and the media provide a constant reminder of that fact. One significant obstacle to achieving this second goal is the fact that effectiveness and realism can work against one another. For example, completely denying the risk of any hazard is likely to be an effective means of preventing fear, but this success at emotional regulation is achieved only by severe distortion of objective risk. Presumably, some appreciation of risk is necessary to motivate individuals to take protective action.

### Strengths and limitations

Perhaps the most notable feature of this study was its timing, which involved data collection just nine days after the WHO declared Zika an international health crisis. This enabled capture of data on public reactions in the early stages of the knowledge diffusion process. It also represents a constraint in that the results may not generalize to later phases of an infectious disease event.

We utilized a sample of women of child-bearing age, all of whom were located in the Southern United States. This had the advantage of assessing persons who were most at risk for the disease and, therefore, most likely to experience fear and the corresponding need for emotional regulation. Because women [47] and younger people [48] generally report higher levels of emotions, the mean values for emotions and, possibly, regulation strategies are probably higher than they would be if the sample were composed of men or older individuals.

Although we examined four different methods of emotion regulation, our sample of strategies was not exhaustive [28]. For example, whereas Webb et al. [19] identify four sub-types of reappraisal, our survey questions, capture only two of them. To the extent possible, future studies might benefit from examining a wider array of strategies. Given the potential for emotional contagion during health crises, consideration of social mechanisms of emotion modulation may be particularly valuable.

Finally, the observed effects for fear and suppression were small by most standards. The estimates provided by the data might be attenuated by methodological factors such as lag time. However, even if the true magnitude of the effects is small, they may be consequential when cumulated over time within individuals or when aggregated across many individuals at one time. More research is needed to assess the pragmatic consequences of the results.

## Conclusions

Because fear of infectious disease has so many consequential and unwanted outcomes, it is rational for citizens and society to be concerned about fear itself. This research focused on whether and how at-risk individuals attempted to manage their fear of Zika at a critical juncture–shortly after Zika was deemed a crisis. Although most of the respondents engaged an emotional regulation strategy and many used multiple methods, none of these efforts was successful at down-regulating fear. Indeed, one strategy–suppression–was demonstrably counterproductive. This study provides valuable insight into individual-level efforts to regulate emotion in a naturally occurring, dynamic communication environment. It also underscores the need to develop interventions to manage fear that are simultaneously effective and realistic.

## Supporting information

S1 Survey Survey instrument. (DOCX)

S1 Data Data to accompany efforts to regulate fear of Zika. (SAV)

## References

[1] Schultz JM, Cooper JL, Baingana F, Oquendo MA, Espinel Z, Althouse BM, et al The role of fear-related behaviors in the 2013–2016 West Africa Ebola Virus Disease outbreak. Current Psychiatry Reports. 2016 11 1;18(11):104 doi: 10.1007/s11920-016-0741-y 27739026 10.1007/s11920-016-0741-y PMC5241909

[2] Yamanis T, Nolan E, Shepler S. Fears and misperceptions of the Ebola response system during the 2014–2015 outbreak in Sierra Leone. PLOS Neglected Tropical Diseases. 2016 10 18;10(10):e0005077 doi: 10.1371/journal.pntd.0005077 27755553 10.1371/journal.pntd.0005077 PMC5068712

[3] Barrett R, Brown PJ. Stigma in the time of influenza: social and institutional responses to pandemic emergencies. Journal of Infectious Diseases. 2008;197(Supplement 1):S34–7. doi: 10.1086/524986 18269326 10.1086/524986

[4] Lempel H, Epstein JM, Hammond RA. Economic cost and health care workforce effects of school closures in the US: Center on Social and Economic Dynamics; 2009 doi: 10.1371/currents.RRN1051 10.1371/currents.RRN1051 PMC2762813 20025205

[5] Tamir M. Why do people regulate their emotions? A taxonomy of motives in emotion regulation. Personality and Social Psychology Review. 2016;20(3):199–222. doi: 10.1177/1088868315586325 26015392 10.1177/1088868315586325

[6] Suls J, Bunde J. Anger, anxiety, and depression as risk factors for cardiovascular disease: the problems and implications of overlapping affective dispositions. Psychological bulletin. 2005;131(2):260 doi: 10.1037/0033-2909.131.2.260 15740422 10.1037/0033-2909.131.2.260

[7] Segerstrom SC, Solomon GF, Kemeny ME, Fahey JL. Relationship of worry to immune sequelae of the Northridge earthquake. Journal of Behavioral Medicine. 1998;21(5):433–450. doi: 10.1023/A:1018732309353 9836130 10.1023/a:1018732309353

[8] Silver RC, Holman EA, Andersen JP, Poulin M, McIntosh DN, Gil-Rivas V. Mental-and physical-health effects of acute exposure to media images of the September 11, 2001, attacks and the Iraq War. Psychological Science. 2013;24(9):1623–1634. doi: 10.1177/0956797612460406 23907546 10.1177/0956797612460406

[9] Romero S, McNeil DG. Zika Virus may be Linked to surge in rare syndrome in Brazil. New York Times [Internet]. 2016 Jan 21; Available from: http://www.nytimes.com/2016/01/22/world/americas/zika-virus-may-be-linked-to-surge-in-rare-syndrome-in-brazil.html?rref=collection%2Fnewseventcollection%2Fzika-virus

[10] McNeil DG. C.D.C. Urges Zika testing for some who are pregnant. New York Times [Internet]. 2016 Jan 19 [cited 2017 Mar 16]; Available from: http://www.nytimes.com/2016/01/20/health/cdc-urges-zika-testing-for-some-who-are-pregnant.html?rref=collection%2Fnewseventcollection%2Fzika-virus

[11] Tavernise S, McNeil DG. Zika virus a global health emergency, W.H.O. says. New York Times [Internet]. 2016 Feb 1; Available from: http://www.nytimes.com/2016/02/02/health/zika-virus-world-health-organization.html?rref=collection%2Fnewseventcollection%2Fzika-virus&action=click&contentCollection=health®ion=stream&module=stream_unit&version=latest&contentPlacement=140&pgtype=collection

[12] Tooby J, Cosmides L. The evolutionary psychology of the emotions and their relationship to internal regulatory variables In: Lewis M, Haviland-Jones JM, Barrett LF, editors. Handbook of emotions. New York: The Guilford Press; 2008 pp. 114–137.

[13] Tomarken AJ, Sutton SK, Mineka S. Fear-relevant illusory correlations: What types of associations promote judgmental bias? Journal of Abnormal Psychology. 1995;104(2):312 doi: 10.1037/0021-843X.104.2.312 7790633 10.1037//0021-843x.104.2.312

[14] Lang PJ, McTeague LM, Bradley MM. RDoC, DSM, and the reflex physiology of fear: A biodimensional analysis of the anxiety disorders spectrum. Psychophysiology. 2016;53(3):336–347. doi: 10.1111/psyp.12462 26877123 10.1111/psyp.12462 PMC4756396

[15] Ekman P. The argument and evidence about universals in facial expressions In: Wagner H, Manstead A, editors. Handbook of social psychophysiology. Chichester: Wiley; 1989 pp. 143–64.

[16] Pérez-Edgar KE, Guyer AE. Behavioral inhibition: Temperament or prodrome? Current Behavioral Neuroscience Reports. 2014;1(3):182–90. doi: 10.1007/s40473-014-0019-9 25101234 10.1007/s40473-014-0019-9 PMC4119720

[17] Oatley K, Johnson-Laird P. Cognitive approaches to emotions. Trends in Cognitive Sciences. 2014;18(3):134–140. doi: 10.1016/j.tics.2013.12.004 24389368 10.1016/j.tics.2013.12.004

[18] Gross JJ. Handbook of emotion regulation New York: The Guilford Press; 2013.

[19] Webb TL, Miles E, Sheeran P. Dealing with feeling: A meta-analysis of the effectiveness of strategies derived from the process model of emotion regulation. Psychological Bulletin. 2012;138(4):775 doi: 10.1037/a0027600 22582737 10.1037/a0027600

[20] Golman R, Hagmann D, Loewenstein G. Information avoidance. Journal of Economic Literature. 2016 doi: 10.1257/jel.20151245

[21] Hovland CI, Janis IL, Kelley HH. Communication and persuasion; psychological studies of opinion change New Haven: Yale University Press; 1953.

[22] Knobloch-Westerwick S, Meng J. Looking the other way: Selective exposure to attitude-consistent and counterattitudinal political information. Communication Research. 2009;36(3): 426–48. doi: 10.1177/0093650209333030

[23] McQueen A, Vernon SW, Swank PR. Construct definition and scale development for defensive information processing: An application to colorectal cancer screening. Health Psychology. 2013; 32(2):190 doi: 10.1037/a0027311 22353026 10.1037/a0027311

[24] Fransen ML, Smit EG, Verlegh PW. Strategies and motives for resistance to persuasion: An integrative framework. Frontiers in Psychology. 2015; 6:1201 doi: 10.3389/fpsyg.2015.01201 26322006 10.3389/fpsyg.2015.01201 PMC4536373

[25] McCrae RR. Situational determinants of coping responses: Loss, threat, and challenge. Journal of Personality and Social Psychology. 1984; 46(4):919 doi: 10.1037/0022-3514.46.4.919 6737200 10.1037//0022-3514.46.4.919

[26] van ‘t Riet J, Ruiter RA. Defensive reactions to health-promoting information: An overview and implications for future research. Health Psychology Review. 2013;7(sup1):S104–S36. doi: 10.1080/17437199.2011.606782

[27] Brans K, Koval P, Verduyn P, Lim YL, Kuppens P. The regulation of negative and positive affect in daily life. Emotion. 2013;13(5):926 doi: 10.1037/a0032400 23731436 10.1037/a0032400

[28] Heiy JE, Cheavens JS. Back to basics: A naturalistic assessment of the experience and regulation of emotion. Emotion. 2014;14(5):878 doi: 10.1037/a0037231 24999913 10.1037/a0037231

[29] Sheppes G, Gross JJ. Is timing everything? Temporal considerations in emotion regulation. Personality and Social Psychology Review. 2011;15(4):319–31. doi: 10.1177/1088868310395778 21233326 10.1177/1088868310395778

[30] Frijda NH. The emotions: Studies in emotion and social interaction Paris: Maison de Sciences de l'Homme; 1986.

[31] Dillard JP, Yang C, Meczkowski E. Defensive reactions to threatening health information: Ebola comes to the United States. Paper presented at National Communication Association; Las Vegas; 2015 Nov 19–22.

[32] Zuwerink Jacks J, Cameron KA. Strategies for resisting persuasion. Basic and Applied Social Psychology. 2003; 25(2):145–61. doi: 10.1207/S15324834BASP2502_5

[33] Gross JJ, Levenson RW. Emotional suppression: physiology, self-report, and expressive behavior. Journal of Personality and Social Psychology. 1993; 64(6):970 8326473 10.1037//0022-3514.64.6.970

[34] Stepper S, Strack F. Proprioceptive determinants of emotional and nonemotional feelings. Journal of Personality and Social Psychology. 1993; 64(2):211 doi: 10.1037/0022-3514.64.2.211

[35] Wegner DM. Ironic processes of mental control. Psychological Review. 1994; 101(1):34 doi: 10.1037/0033-295X.101.1.34 8121959 10.1037/0033-295x.101.1.34

[36] Faul F, Erdfelder E, Buchner A, Lang A-G. Statistical power analyses using G* Power 3.1: Tests for correlation and regression analyses. Behavior Research Methods. 2009; 41(4):1149–60. doi: 10.3758/BRM.41.4.1149 19897823 10.3758/BRM.41.4.1149

[37] Carmines EG, McIver JP. Analyzing models with unobserved variables: Analysis of covariance structures. Social measurement: Current issues. 1981:65–115.

[38] Hu L-t, Bentler PM. Fit indices in covariance structure modeling: Sensitivity to underparameterized model misspecification. Psychological methods. 1998; 3(4):424 doi: 10.1037/1082-989x.3.4.424

[39] Hu L-t, Bentler PM. Cutoff criteria for fit indexes in covariance structure analysis: Conventional criteria versus new alternatives. Structural equation modeling: a multidisciplinary journal. 1999; 6(1):1–55. doi: 10.1080/10705519909540118

[40] Little TD. Longitudinal structural equation modeling: Guilford Press; 2013.

[41] Cheung GW, Rensvold RB. Evaluating goodness-of-fit indexes for testing measurement invariance. Structural equation modeling. 2002; 9(2):233–55.

[42] Brans K, Verduyn P. Intensity and duration of negative emotions: Comparing the role of appraisals and regulation strategies. PLOS ONE. 2014; 9(3):e92410 doi: 10.1371/journal.pone.0092410 24670979 10.1371/journal.pone.0092410 PMC3966809

[43] Yang C, Dillard JP, Li R. Understanding fear of Zika: Personal, interpersonal, and media influences. Risk Analysis. 2018 2 02 doi: 10.1111/risa.12973 29392760 10.1111/risa.12973

[44] Gollob HF, Reichardt CS. Taking account of time lags in causal models. Child Development. 1987;58:80–92. doi: 10.2307/1130293 3816351

[45] Lederman SA, Rauh V, Weiss L, Stein JL, Hoepner LA, Becker M, et al The effects of the World Trade Center event on birth outcomes among term deliveries at three lower Manhattan hospitals. Environmental Health Perspectives. 2004:1772–8. doi: 10.1289/ehp.7348 15579426 10.1289/ehp.7348 PMC1253672

[46] Smits L, Krabbendam L, De Bie R, Essed G, Van Os J. Lower birth weight of Dutch neonates who were in utero at the time of the 9/11 attacks. Journal of Psychosomatic Research. 2006;61(5):715–7. doi: 10.1016/j.jpsychores.2006.04.020 17084151 10.1016/j.jpsychores.2006.04.020

[47] Campbell A, Coombes C, David R, Opre A., Grayson L, & Muncer S. (2016). Sex differences are not attenuated by a sex-invariant measure of fear: The situated fear questionnaire. Personality and Individual Differences. 2016; 97:210–219. doi: 10.1016/j.paid.2016.03.049

[48] Charles ST, Reynolds CA, Gatz M. Age-related differences and change in positive and negative affect over 23 years. Journal of Personality and Social Psychology. 2001; 80(1):136 doi: 10.1037/0022-3514.80.1.136 11195886

