# Supplementary material for: Self-regulation of emotional responses to Zika: Spiral of fear
Source: PLoS One. 2018 Jul 10;13(7):e0199828. doi: 10.1371/journal.pone.0199828 (PMC6039018; doi:10.1371/journal.pone.0199828)
Supplement: S1 Survey — (DOCX) [file pone.0199828.s001.docx]

**Shortened Version of the Questionnaire**

**Gender**

What is your gender?

1 = Male, 2 = Female

**Age**

What is your age in years?

**Race/Ethnicity**

Which of the following categories do you identity with? Check all that apply to you.

1= White, 2 = Hispanic/Latino, 3 = Black / African American, 4 = Native American / American Indian, 5 = Asian / Pacific Islander, 6 = Other

**Issue involvement** (0 = No, 1 = Yes)

I expect that I will become pregnant in the next two years.

I am pregnant now.

**Avoidance** (Likert-type scale, 1 = *Strongly disagree*, 7 = *Strongly agree*)

I actively avoided news about Zika.

*I decided not to get into conversations about Zika.

*I kept my attention on the important things in life.

I avoided situations where I would hear about Zika.

I stayed away from people or media who were likely to discuss Zika.

**Reappraisal** (Likert-type scale, 1 = *Strongly disagree*, 7 = *Strongly agree*)

*I focused on the idea that Zika might hurt others, but it won’t get me or the people that I care about.

*I reminded myself that the chances of actually being harmed by Zika are close to zero.

I remembered that life is full of risks: You have to accept that fact.

*There is really nothing that I can to do to change the odds that I or someone I care about will contract Zika.

*I thought that life is too busy to worry about one more risk.

I reminded myself to accept that which I cannot change.

**Suppression** (Likert-type scale, 1 = *Strongly disagree*, 7 = *Strongly agree*)

I made an effort not to think about Zika.

I tried to avoid thinking about Zika.

I tried to tamp down my feelings about Zika.

*I ignored my feelings about Zika.

*I tried to stay positive.

**Counterarguing** (Likert-type scale, 1 = *Strongly disagree*, 7 = *Strongly agree*)

I remembered that most of what we are hearing about Zika is blown out of proportion.

I reminded myself that people are making too big a deal out of Zika.

I thought that most of what I hear about Zika is exaggerated.

*I thought that the health agencies don’t really know what they are talking about.

I thought that the media are exaggerating the problem.

* Items with asterisks were identified via factor analysis as suboptimal. They were not included in the final measures.
